# Supplementary material for: Architecture of the ESCPE-1 membrane coat
Source: Nat Struct Mol Biol. 2023 Jun 15;30(7):958–69. doi: 10.1038/s41594-023-01014-7 (PMC10352136; doi:10.1038/s41594-023-01014-7)
Supplement: Supplementary file 1 — Reporting Summary [file 41594_2023_1014_MOESM1_ESM.pdf]

nature portfolio

Corresponding author(s): Aitor Hierro
Daniel Castaño-Díez
Juan S. Bonifacino

Last updated by author(s): Mar 30, 2023

Reporting Summary

Nature Portfolio wishes to improve the reproducibility of the work that we publish. This form provides structure for consistency and transparency in reporting. For further information on Nature Portfolio policies, see our Editorial Policies and the Editorial Policy Checklist.

Statistics

For all statistical analyses, confirm that the following items are present in the figure legend, table legend, main text, or Methods section.

n/a	Confirmed	
☐	☒	The exact sample size (*n*) for each experimental group/condition, given as a discrete number and unit of measurement
☐	☒	A statement on whether measurements were taken from distinct samples or whether the same sample was measured repeatedly
☐	☒	The statistical test(s) used AND whether they are one- or two-sided<br>*Only common tests should be described solely by name; describe more complex techniques in the Methods section.*
☒	☐	A description of all covariates tested
☒	☐	A description of any assumptions or corrections, such as tests of normality and adjustment for multiple comparisons
☐	☒	A full description of the statistical parameters including central tendency (e.g. means) or other basic estimates (e.g. regression coefficient) AND variation (e.g. standard deviation) or associated estimates of uncertainty (e.g. confidence intervals)
☐	☒	For null hypothesis testing, the test statistic (e.g. *F*, *t*, *r*) with confidence intervals, effect sizes, degrees of freedom and *P* value noted<br>*Give P values as exact values whenever suitable.*
☒	☐	For Bayesian analysis, information on the choice of priors and Markov chain Monte Carlo settings
☒	☐	For hierarchical and complex designs, identification of the appropriate level for tests and full reporting of outcomes
☒	☐	Estimates of effect sizes (e.g. Cohen's *d*, Pearson's *r*), indicating how they were calculated

Our web collection on statistics for biologists contains articles on many of the points above.

Software and code

Policy information about availability of computer code

Data collection	• Crystallographic native and derivative data sets were collected with the software MxCuBE at XALOC beamline in ALBA (Cerdanyola del Valles, Spain) using a Pilatus 6M detector.<br>• High-resolution cryoET data was obtained with Serial-EM 4.0 software in an FEI Titan Krios G3 microscope, coupled with Gatan K2 Summit direct detector.<br>• For live-cell imaging, images were acquired with Zeiss ZEN Black 2.3 software on a Zeiss LSM780 or Zeiss LSM880 inverted confocal laser scanning microscope fitted with a Plan-Apochromat 63X (NA=1.4) objective (Carl Zeiss).<br>• For immunofluorescence microscopy, images were acquired using Zeiss ZEN Black 2.3 software (Carl Zeiss) on a confocal microscope (LSM710 or LSM880; Carl Zeiss) with an oil-immersion 63×/1.40 NA Plan-Apochromat Oil DIC M27 objective lens (Carl Zeiss).
Data analysis	• X-ray diffraction images were indexed, integrated and scaled using XDS (BUILT=20170923) or MOSFLM/SCALA 7.2.2. Heavy atom positions were identified using SHELXC/D as implemented in autoSHARP 3.10.8. Density modification was done using SOLOMON as implemented in autoSHARP 3.10.8. Refinement was done with PHENIX 1.18 and manual model building with COOT 0.8.9.<br>• Cryo-tomography tilt series alignment and tomogram reconstruction was done using IMOD 4.5.0. Sub-tomogram averaging was done with the Dynamo software.<br>• ITC data was fitted and integrated employing the MicroCal PEAQ-ITC software VPViewer2000, from Malvern Panalytical. Final graphs were prepared using Origin 7 ITC software (MicroCal).<br>• Binding energy per residue was estimated with resEnergy pyDock.<br>• Fluorescence microscopy images were processed in ImageJ/Fiji 1.52k (https://fiji.sc). Statistical analyses were performed using Prism Software 9.3.1 (GraphPad).

For manuscripts utilizing custom algorithms or software that are central to the research but not yet described in published literature, software must be made available to editors and reviewers. We strongly encourage code deposition in a community repository (e.g. GitHub). See the Nature Portfolio guidelines for submitting code & software for further information.

Data

Policy information about availability of data

All manuscripts must include a data availability statement. This statement should provide the following information, where applicable:

- Accession codes, unique identifiers, or web links for publicly available datasets
- A description of any restrictions on data availability
- For clinical datasets or third party data, please ensure that the statement adheres to our policy

Atomic coordinates and structure factors of the crystallographic complexes are available in the Protein Data Bank (PDB) with accession codes 8A1G and 8ABQ (Table 1). Cryo-ET structures and representative tomograms have been deposited in the Electron Microscopy Data Bank (EMDB) with accession code EMD-15413, and the associated PDB 8AFZ (Table 2). Dose-weighted tilt series are available in the Electron Microscopy Public Image Archive (EMPIAR) under the accession code 11484. Additional data that support the findings of this study are available from the corresponding authors on request.
The matlab scripts used to compute the neighborhood analysis have been implemented in Dynamo 4.9 (freely available for download at dynamo-em.org) and its functionalities can be accessed through the command dpktbl.neighborhood.analize.
Validation reports are included in Supplementary Information. Additional data that support the findings of this study are available from the corresponding authors on request.

Human research participants

Policy information about studies involving human research participants and Sex and Gender in Research.

Reporting on sex and gender	No human subjects involved in this study
Population characteristics	Not applicable
Recruitment	Not applicable
Ethics oversight	Not applicable

Note that full information on the approval of the study protocol must also be provided in the manuscript.

Field-specific reporting

Please select the one below that is the best fit for your research. If you are not sure, read the appropriate sections before making your selection.

☒ Life sciences ☐ Behavioural & social sciences ☐ Ecological, evolutionary & environmental sciences

For a reference copy of the document with all sections, see nature.com/documents/nr-reporting-summary-flat.pdf

Life sciences study design

All studies must disclose on these points even when the disclosure is negative.

Sample size	For sub-tomogram averaging, all intact tubes with a minimum length of ~160 nm and no extreme curvatures were manually traced along their center in all tomograms. In total, 180 tubes were used for further processing. Sample size was was predetermined by the microscope allocation time. Data was sufficient to achieve the reported resolutions and build the molecular models. The number of replicates for the functional assays are stated in the figure legends.
Data exclusions	Sub-tomogram outlier exclusion: Sub-volumes that fulfilled at least one of the following criteria were excluded from further processing: (a) Extreme radius: Sub-volume coordinate too close or too far from the tube center. (b) Extreme angle: Normal vector of sub-volume differs too much from normal vector of tube surface. (c) Missing neighbor: Sub-volume has no neighboring particles on either of its tips. (d) Low cross correlation: Sub-volume has a low cross-correlation to the reference.
Replication	Liposome tubulation, liposome flotation and ITC assays were repeated at least twice and all attempts at replication were successful. 'In cellulo' functional studies were repeated as indicated in the figure legends.
Randomization	For immunofluorescence analysis, images were randomly acquired at the confocal microscope for each set of experiments. For cryo-ET, the most promising areas for data collection were selected from low magnification images.
Blinding	Imaging of liposome tubulation reactions was performed by someone blind to the composition of the reactions. All light microscopy experiments have been measured by blinded personnel when required manual intervention.

Reporting for specific materials, systems and methods

We require information from authors about some types of materials, experimental systems and methods used in many studies. Here, indicate whether each material, system or method listed is relevant to your study. If you are not sure if a list item applies to your research, read the appropriate section before selecting a response.

Materials & experimental systems

n/a	Involved in the study
☐	☒ Antibodies
☐	☒ Eukaryotic cell lines
☒	☐ Palaeontology and archaeology
☒	☐ Animals and other organisms
☒	☐ Clinical data
☒	☐ Dual use research of concern

Methods

n/a	Involved in the study
☒	☐ ChIP-seq
☒	☐ Flow cytometry
☒	☐ MRI-based neuroimaging

Antibodies

Antibodies used	The following primary antibodies were used for immunoblotting and/or immunofluorescence microscopy: rabbit anti-SNX1 (1:2,000 dilution) (Atlas Antibodies, HPA047373), rabbit anti-SNX2 (1:2,000 dilution) (Atlas Antibodies, HPA037400), rabbit anti-SNX5 (1:2,000 dilution) (Abcam, ab180520), rabbit anti-SNX6 (1:2,000 dilution) (Atlas Antibodies, HPA049374), rabbit anti-EEA1 (1:100 dilution) (Cell Signaling Technology, C45B10), mouse anti-CI-MPR (1:200 dilution) (Abcam, 2G11), chicken anti-GFP (1:500 dilution) (Thermo Fisher Scientific, A10262), mouse HRP-conjugated anti-α-tubulin (1:10,000) (Santa Cruz Biotechnology, DM1A), rat anti-HA epitope (1:10,000) (Roche, 3F10). HRP-conjugated goat anti-rabbit (1:5,000 dilution) (Jackson ImmunoResearch, AB_2313567), Alexa Fluor 594 donkey anti-rat IgG (1:1,000 dilution) (Thermo Fisher Scientific, A21209), Alexa Fluor 555 donkey anti-mouse IgG (1:1,000 dilution) (Thermo Fisher Scientific, A31570), Alexa Fluor 488 donkey anti-rabbit IgG (1:1,000 dilution) (Thermo Fisher Scientific, A21206) and Alexa Fluor 488 goat anti-chicken IgG (1:1,000 dilution) (Thermo Fisher Scientific, A11039).
Validation	All primary antibodies were validaded by the vendor for immunoblotting and/or immunofluorescence. The validation statement can be found on the corresponding manufacture's website.

Eukaryotic cell lines

Policy information about cell lines and Sex and Gender in Research

Cell line source(s)	• HT1080, American Type Culture Collection CCL-121<br>• HEK293T, American Type Culture Collection CRL-3216
Authentication	The expression (or lack thereof) of target proteins in WT and KO HT1080 KO cells was confirmed by immunoblotting with specific antibodies.
Mycoplasma contamination	The cell lines were not tested for mycoplasma contamination.
Commonly misidentified lines (See ICLAC register)	The HT1080 cells used in this study have not been reported to be contaminated by any other cells according to https://iclac.org/databases/cross-contaminations/. No commonly misidentified cell lines were used in the study.
