# Supplementary figures and images for: Architecture of the ESCPE-1 membrane coat
Source: Nat Struct Mol Biol. 2023 Jun 15;30(7):958–69. doi: 10.1038/s41594-023-01014-7 (PMC10352136; doi:10.1038/s41594-023-01014-7)

Figure 1a

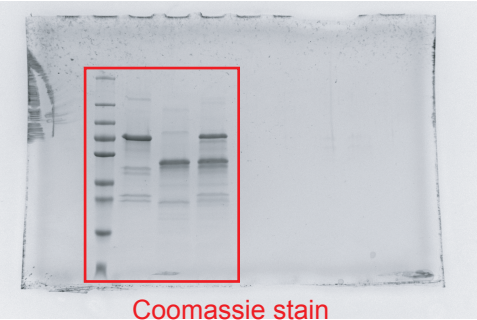

Supplement: Supplementary file 5 — Unprocessed gels. [file 41594_2023_1014_MOESM5_ESM.pdf]

Figure 2a

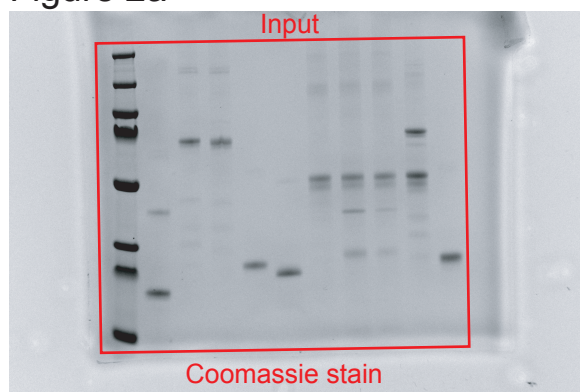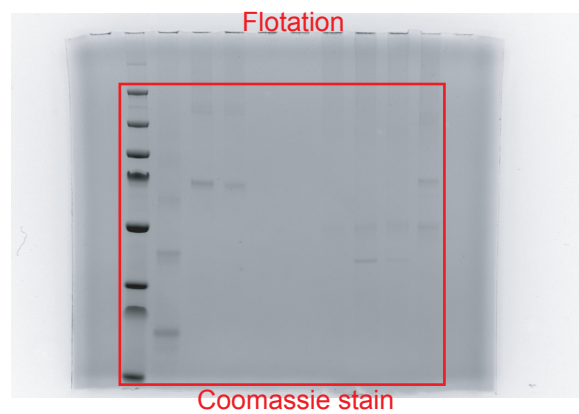

Figure 2b SNX5

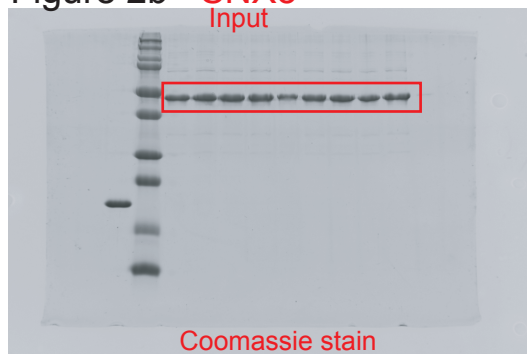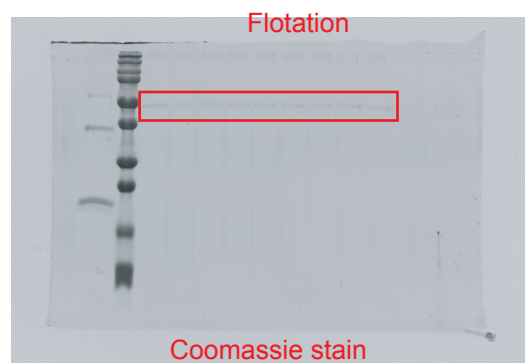

Figure 2b SNX1

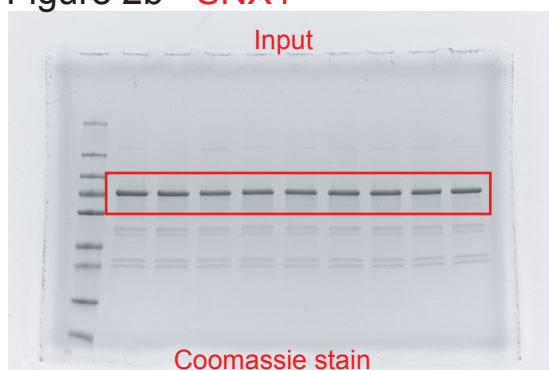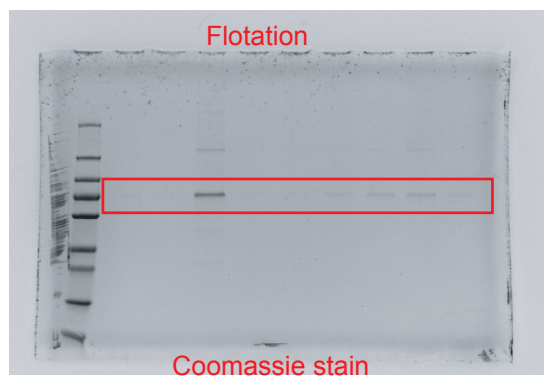

Figure 2b SNX1 PX domain

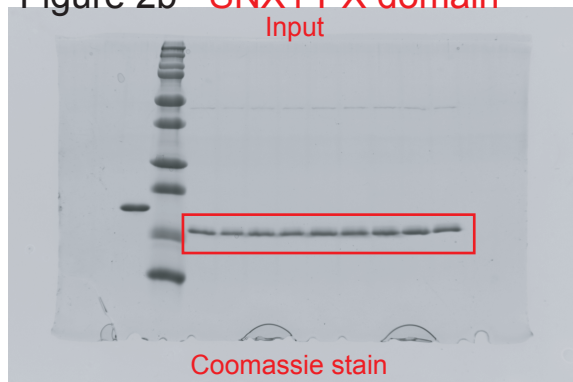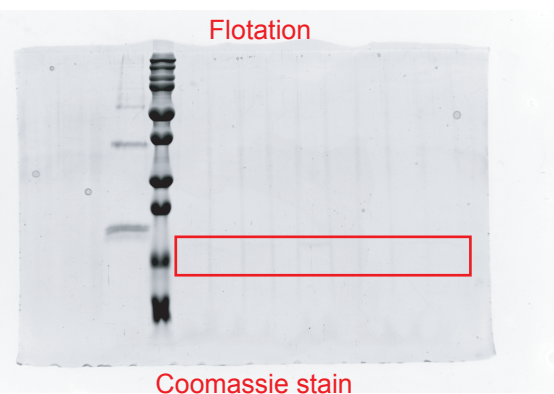

Figure 2c

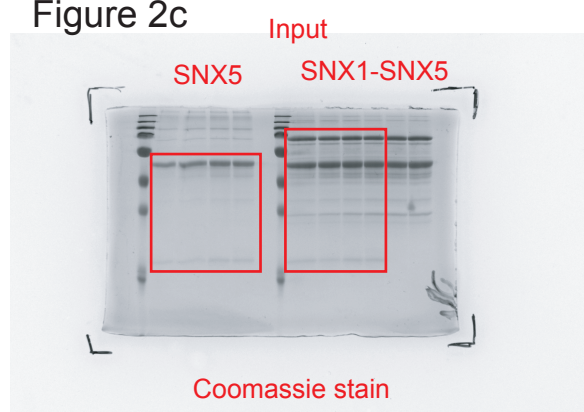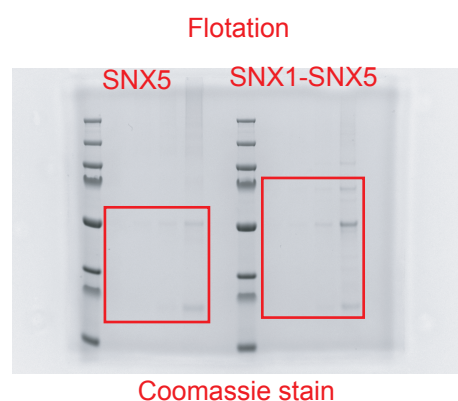

Supplement: Supplementary file 7 — Unprocessed gels. [file 41594_2023_1014_MOESM7_ESM.pdf]

Figure 5a

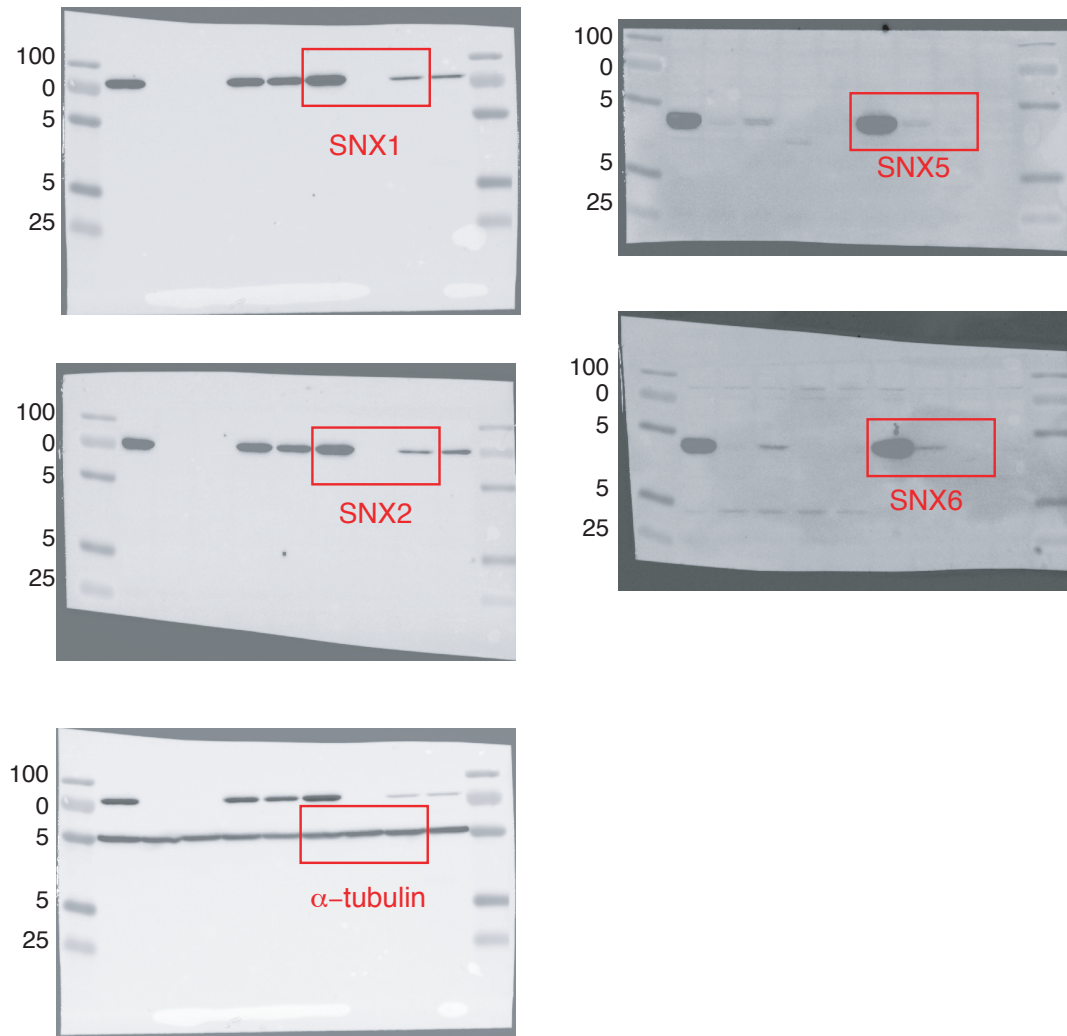

Figure 5c

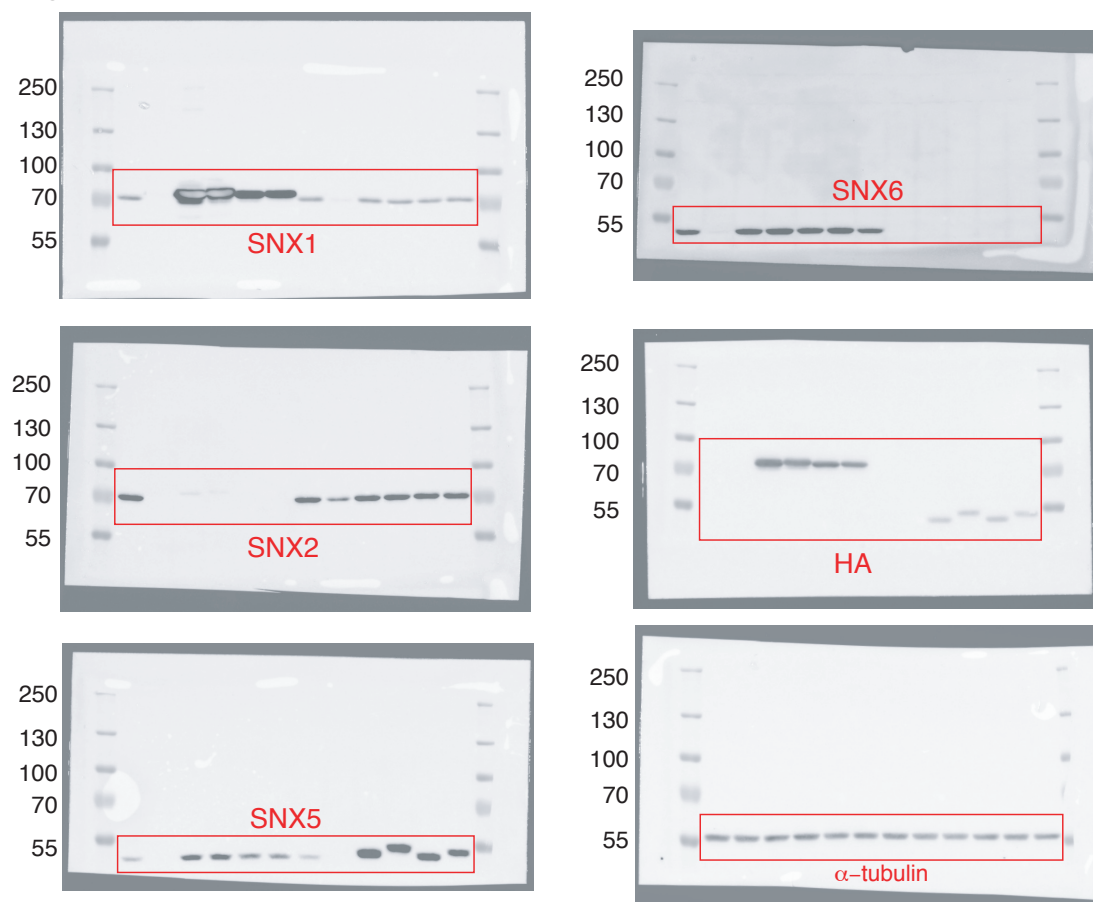

Supplement: Supplementary file 8 — Unprocessed gels and western blots. [file 41594_2023_1014_MOESM8_ESM.pdf]

Extended Data Figure 4b

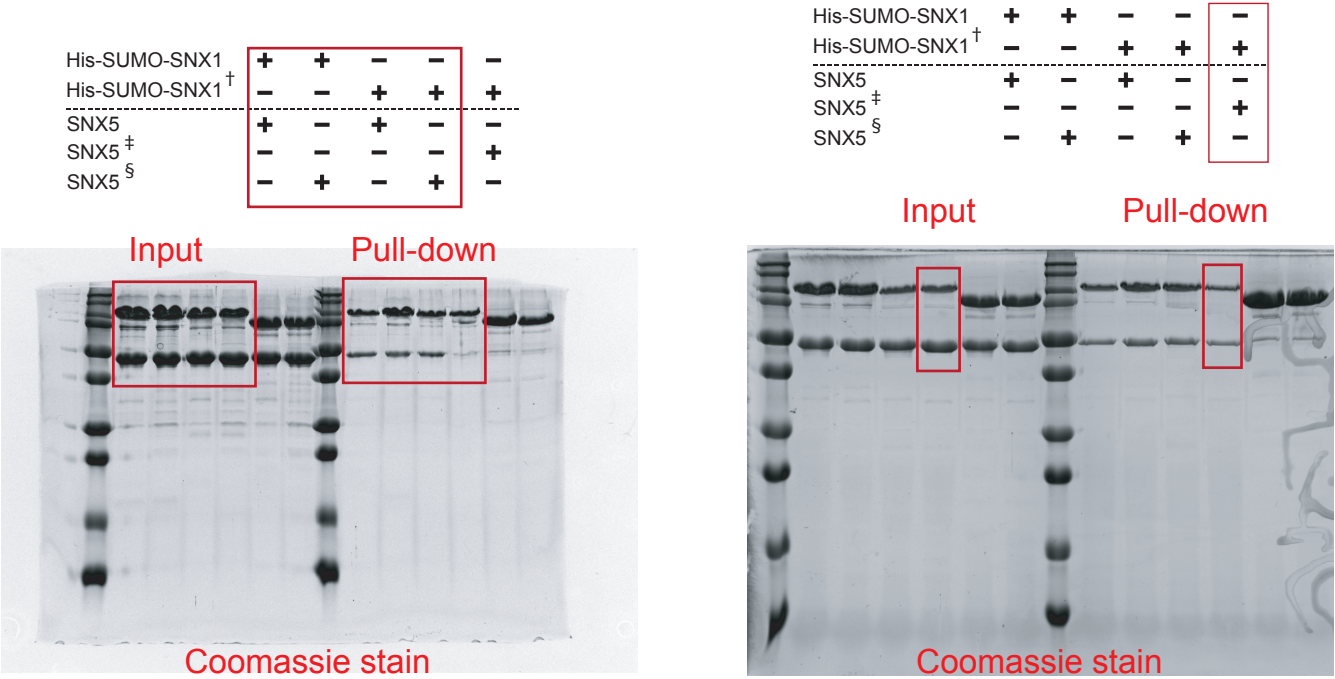

Extended Data Figure 4c

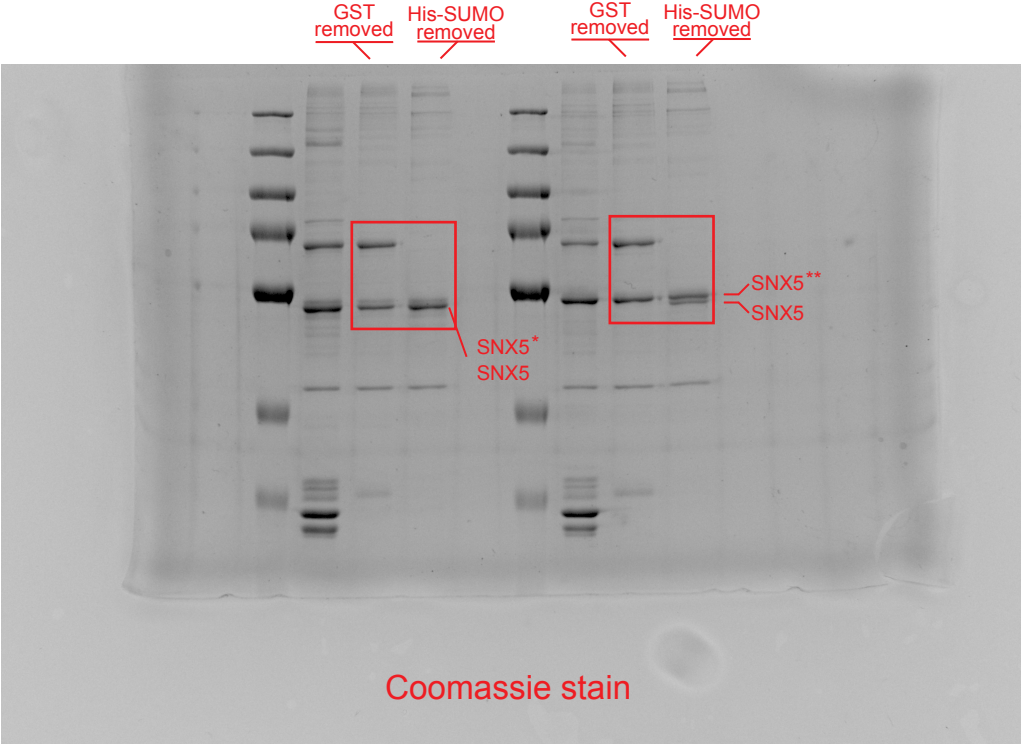

Supplement: Supplementary file 11 — Unprocessed gels. [file 41594_2023_1014_MOESM11_ESM.pdf]
